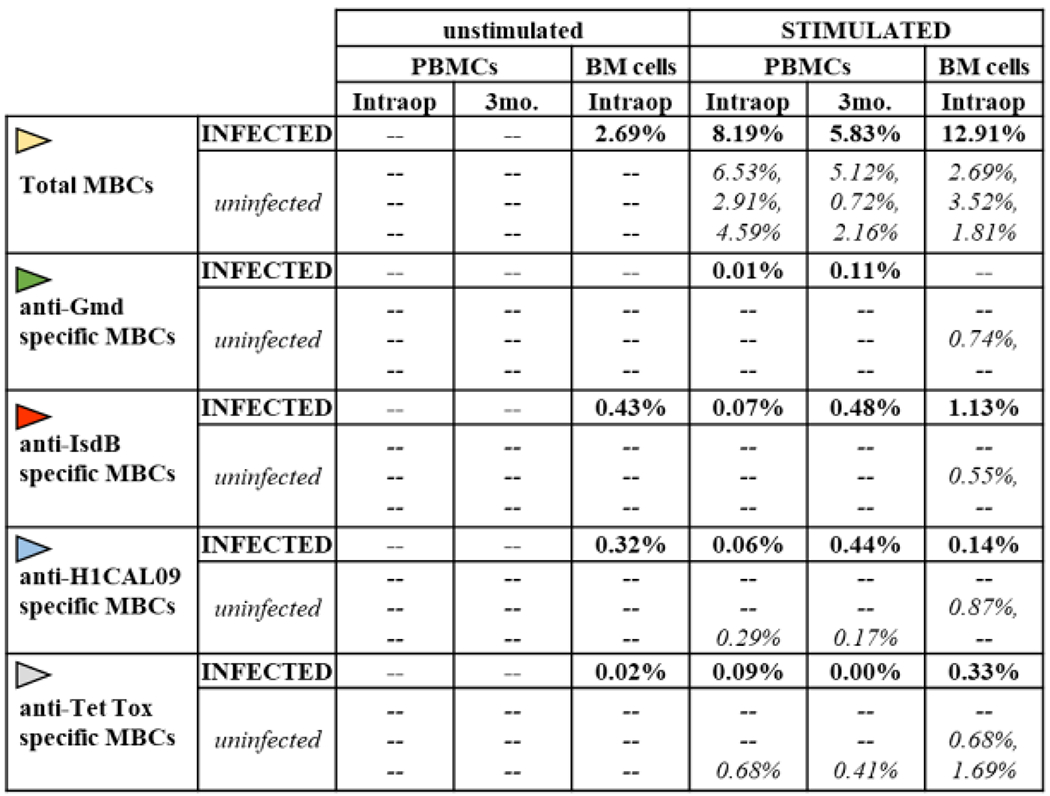# Anti-IsdB Antibody-Secreting Cells Found in S. aureus-Infected Periprosthetic Bone Marrow: Case Series of Total Hip Arthroplasty Patients

**DOI:** 10.18103/mra.v14i1.7191

**Published:** 2026-01

**Authors:** John R. Owen, Gowrishankar Muthukrishnan, David J. Topham, John L. Daiss, Edward M. Schwarz, Stephen L. Kates

**Affiliations:** 1 Department of Orthopaedic Surgery, Virginia Commonwealth University, Richmond, VA, USA; 2 Center for Musculoskeletal Research, University of Rochester Medical Center, Rochester, NY, USA; 3 Department of Microbiology & Immunology, University of Rochester Medical Center, Rochester, NY, USA

**Keywords:** Orthopaedic Infections, Immunoassay, *Staphylococcus aureus*, Osteomyelitis

## Abstract

Staphylococcus aureus periprosthetic joint infection is notoriously difficult to treat and is often associated with septic death. Results from the V710 Phase IIB/III clinical trial and subsequent research have demonstrated that non-neutralizing antibodies against the iron-regulated surface determinant protein B (IsdB) facilitate bacterial entry into leukocytes, generating “Trojan horse” leukocytes and the dissemination of surgical-site infections. In contrast, anti-glucosaminidase (Gmd) antibodies, which mediate opsonophagocytosis of bacterial clusters, are associated with protection from S. aureus osteomyelitis in both mice and humans. To further test whether anti-IsdB antibodies are pathogenic and anti-Gmd antibodies are protective in infected patients, we performed a clinical pilot study of three healthy patients undergoing primary total hip arthroplasty and a patient undergoing revision arthroplasty for S. aureus infection to assess the feasibility of quantifying anti-IsdB and anti-Gmd antibody-secreting cells in blood and periprosthetic bone marrow. We also assessed anti-IsdB and anti-Gmd antibody levels in serum, blood plasma, bone marrow plasma, and cell culture supernatant collected after six days with and without a previously established memory B-cell stimulation cocktail. The frequencies of memory B-cells and antibody-secreting plasmablasts were measured using IgG ELISpot. Consistent with our hypothesis, we found large numbers of anti-IsdB antibody-secreting cells in unstimulated periprosthetic bone marrow from the infected patient, which increased with stimulation. However, these cells were only detectable in peripheral blood mononuclear cells following stimulation. Furthermore, anti-Gmd antibody-secreting cells were undetectable in the bone marrow of this infected patient at the time of revision surgery. This patient also had very high titers of anti-IsdB antibodies in serum, blood plasma, and bone marrow plasma. Anti-IsdB and anti-Gmd antibody-secreting cells were only detected in bone marrow for one of the uninfected patients. Collectively, the results are consistent with the hypothesis that anti-IsdB antibodies are associated with disease and related to the presence of IsdB-specific memory B-cells and plasmablasts. This case series demonstrates the utility of ELISpot to assess local vs. systemic antigen-specific humoral responses in total hip arthroplasty patients and support a larger study to test the hypothesis that anti-IsdB antibody-secreting cells proximal to S. aureus infected implants are associated with recalcitrant infections and septic death.

## Introduction

The incidence of *Staphylococcus aureus* periprosthetic joint infections appears to be largely unaffected by continued improvements in surgical techniques and aseptic practice^1,2^, remaining at a rate of 1%-2%^3,4^. Once an infection occurs, eradication is very difficult to achieve, reinfection rates can be as high as 40%^5,6^ at great financial costs to the healthcare system^7^, and roughly 10% of these infection cases result in septic death^8^. All attempts to develop vaccines against *Staphylococcus aureus* have failed^9,10^. Results from the V710 Phase IIB/III clinical trial^11^ and subsequent research^12^ demonstrated that non-neutralizing antibodies against the iron-regulated surface determinant protein B (IsdB) facilitate bacterial entry into leukocytes to generate “Trojan horse” leukocytes and dissemination of surgical-site infection^8,13^. In contrast, anti-glucosaminidase (Gmd) antibodies^14^, which mediate opsonophagocytosis of bacterial clusters^15^, are associated with protection from *S. aureus* osteomyelitis in mice^16^ and humans^15–18^. To further test this immune proteome hypothesis^19,20^ that anti-IsdB antibodies are pathogenic and anti-Gmd antibodies are protective in periprosthetic joint infection patients^21,22^, we performed a clinical pilot study to assess the feasibility of quantifying anti-IsdB and anti-Gmd antibody-secreting cells systemically in blood and locally in periprosthetic bone marrow from total hip replacement patients via ELISpot assays, while also assessing anti-IsdB and anti-Gmd antibody levels via Luminex assays^23^ of blood, bone marrow, and supernatant collected during ELISpot assay preparation. Here, we present a case series of three healthy patients undergoing primary total hip arthroplasty and a patient undergoing revision arthroplasty for *S. aureus* periprosthetic joint infection.

## Materials and Methods

### HUMAN SUBJECTS:

All human subject research was performed with informed consent under IRB-approved protocols. One infected subject (44-year-old female), and three uninfected subjects (one 65-year-old female, and two males aged 49 and 64) are reported in this case series.

### TISSUE HARVEST AND STORAGE:

Bone marrow was collected at the time of surgery from the “safe zone” of the acetabulum into three heparinized tubes for cell processing as previously described^24^. Peripheral blood was collected in one serum separation tube and three heparinized tubes at the time of surgery, and at follow-up three months post-surgery, as previously described^25^. After centrifugation, serum was collected from the serum separation tube and all but about 0.5ml plasma per tube was collected from blood and bone marrow heparinized tubes and stored in aliquots at −80°C. Approximately 0.5ml of plasma was left in each heparinized tube above underlying blood or bone marrow components to avoid accidentally removing cells.

The remaining plasma and underlying bone marrow components were diluted in a 50ml conical tube with PBS at a ratio of ≤1:4 based on the initial bone marrow volume but not exceeding a total diluted volume of 35ml. To remove bone fragments, the diluted bone marrow was poured through a 100μl cell strainer (Falcon 352360) into another 50ml conical tube and then a 40μl cell strainer. (Fisher 22363547) into a third 50ml conical tube to remove adipose fat cells. The remaining plasma and underlying blood components were then diluted in a 50ml conical tube with PBS at a 1:2 ratio, based on the initial blood volume, but not over 35ml total diluted volume. The diluted blood and the diluted/filtered bone marrow were then poured into separate 50ml Leucosep tubes (227290P, Greiner Bio-One) containing 15ml lymphocyte separating media (25-072-CV, Corning) in the base of each tube. Leucosep tubes were then centrifuged to separate out the “buffy coat” containing peripheral blood mononuclear cells (PBMCs) or bone marrow cells. All but 10ml of liquid, which contained the “buffy coat,” was removed by serological pipette and discarded.

The approximately 10ml of liquid remaining containing PBMCs or bone marrow cells was then poured into separate 15ml conical tubes. PBS was added to these tubes up to the 14ml level in each tube, the cells were suspended by inversion, and the tubes were centrifuged at 4°C, 400g, 12min, accel=9, brake=9. Afterward, supernatant was removed via serological pipette without disturbing the cell pellet and 3ml of ACK lysing buffer (A10492-01, Gibco) was added and the cell pellet resuspended for 4min to lyse red blood cells. PBS was then added up to the 14ml level and centrifugation was repeated, followed by supernatant removal. ACK lysing buffer was applied a second time with cell suspension but for only 2 minutes. A final wash was then performed by adding PBS again to the 14ml level, followed by centrifugation and supernatant removal. The PBMC and bone marrow cell pellets were then resuspended in 2ml of CryoStor^®^ CS10 cell freezing media (100-1061, Stem Cell Technologies, Inc.). Cell density was measured via Luna cell counter and enough CryoStor^®^ media was added to achieve a final cell density of about 3x10^6^ cells per ml. PBMCs and bone marrow cell aliquots of about 3x10^6^ cells each were then placed in a Corning CoolCell container to control freezing in a −80°C freezer. We also saved a couple bone marrow cell aliquots of about 1x10^6^ cells each for future cell sequencing studies. After 24hrs, the aliquots were transferred to storage in liquid nitrogen vapor (Thermo Scientific, Cryoextra CE8100 Series Liquid Nitrogen Storage System).

### ELISPOT ASSAYS:

**Figure 1:**
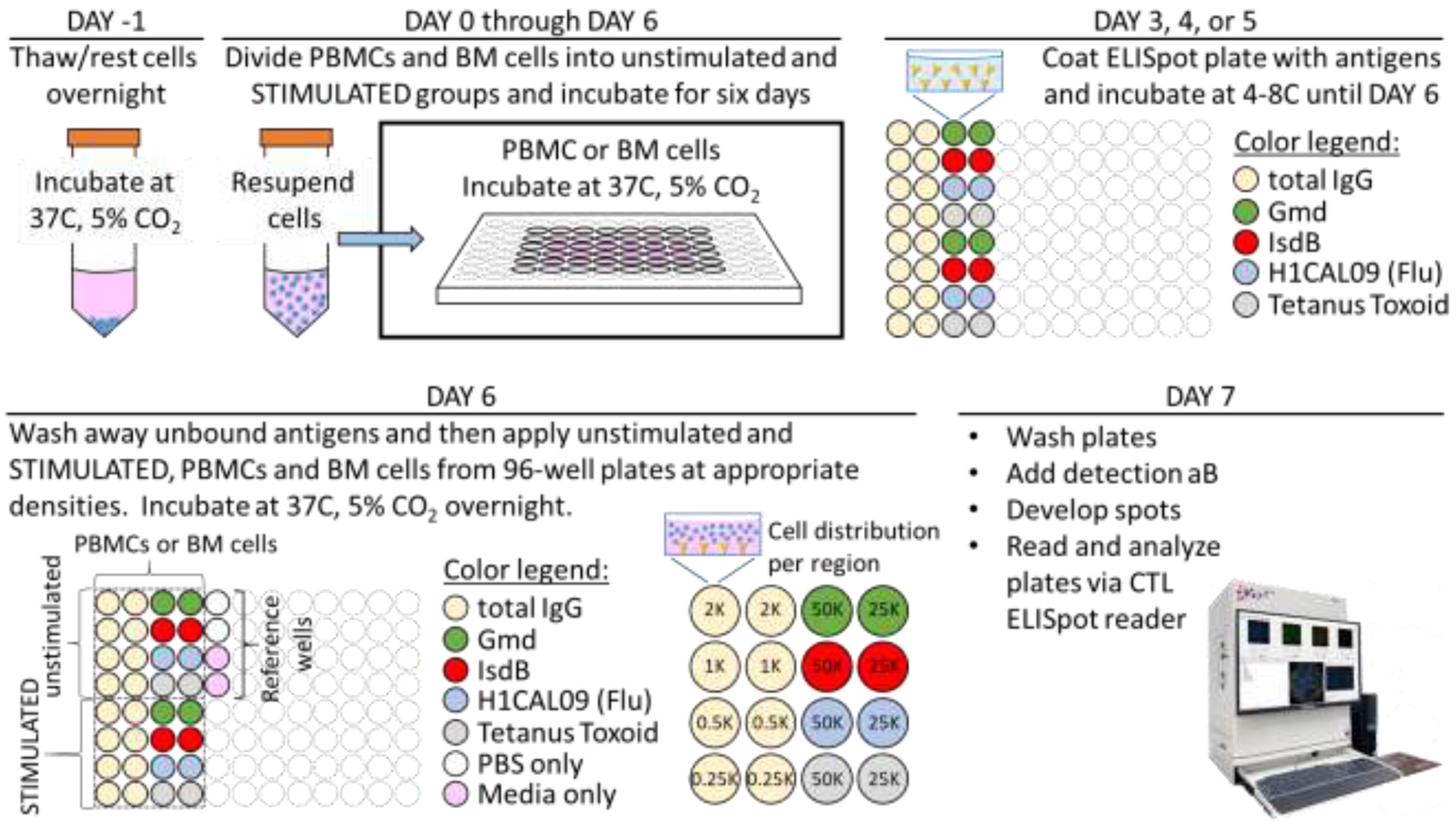
Schematic of the experimental design of the primary PBMC and bone marrow (BM) cell culture, stimulation, ELISpot assay and analysis. The required steps to assess cellular response to specific antigens spans eight days from start-to-finish.

ELISpot assays were performed to quantify anti-IsdB and anti-Gmd antibody-secreting cells systemically in blood and locally in periprosthetic bone marrow. One vial each of previously stored PBMCs and bone marrow cells (approximately 3x10^6^ cells per vial) were thawed and prepared during a seven-day process culminating in ELISpot assay and analysis as previously described for PBMCs^26,27^ but adapted here to include bone marrow cells and to investigate *S. aureus* antigens of interest (IsdB and Gmd). Before beginning the seven-day process necessary for ELISpot assay, cells were thawed on Day -1 and allowed to rest overnight in cell culture media at 37°C, 5% CO_2_. (Figure 1)

On Day 0, cell densities of PBMCs and bone marrow cells were each adjusted to 1x10^6^ cells/ml and each of the cell mixtures was divided into two groups: one half for incubation without stimulation (unstimulated), and one half for incubation with stimulation (STIMULATED). As previously described^26,27^, 2x cocktails with and without stimulation components, were added at a 1:2 ratio to the unstimulated and STIMULATED groups bringing cell densities to 5x10^5^ cells/ml. The cell mixtures were then distributed 200μl per well to their respective U-bottom 96-well plates, (Corning, COSTAR cat no. 3799) across a group of wells. PBS (200μl per well) was placed in wells around the periphery of the group of wells to minimize evaporation of media from the wells containing cells. These four plates (unstimulated PBMCs, STIMULATED PBMCs, unstimulated bone marrow cells, and STIMULATED bone marrow cells) were then covered and incubated at 37°C, 5% CO_2_ for six days.

One to three days before completing the six days of incubation, typically on Day 5, three ELISpot plates (MSIPS4W10, Millipore), one for each cell type/timepoint, were coated in specific wells by previously determined antigen amounts: total IgG (0.25μg/well), Gmd (0.5μg/well), IsdB (0.25μg/well), H1CAL09 (0.25μg/well), and Tetanus Toxoid (0.5μg/well). These plates were then incubated at 4-8°C until Day 6. Total IgG is used to determine the percentage of memory B-cells present in PBMCs and bone marrow cells. IsdB and Gmd are used to determine the percentage of those memory B-cells that produce antibodies against IsdB and Gmd. H1CAL09 (a flu antigen) and Tetanus Toxoid are used as non-*S. aureus* positive reference antigens for which most people will have specific antibodies.

On Day 6, cell mixtures were removed from the 96-well plates by pipette and centrifuged to collect antibody-rich supernatant aliquots that were frozen (−80°C) for future Luminex testing. Cells were then washed twice with culture media, resuspended in media, and then distributed as 100μl per well at appropriate densities to corresponding ELISpot plate wells. A replicated four-fold series dilution from 2,000 to 250 cells per well was applied across the total IgG wells and the remaining cells were distributed as a two-fold dilution (approximately 50K/25K cells on average) across the columns dedicated to specific antigens. The plates were then incubated overnight at 37°C, 5% CO_2_.

On Day 7, following the established protocol, the plates were washed to remove all cells and unbound antibodies. An anti-IgG detection antibody (Jackson Immuno Research Labs 109-055-008) was applied, and the plates were incubated for 2hrs in the dark at room temperature. The plates were then washed and soaked in PBST for 45min. Afterward, PBST was removed and an alkaline phosphatase substrate (Vector Laboratories SK-5300) was applied. Plates were then incubated at room temperature until sufficient spot development. Afterward, the plates were gently washed under cold tap water and allowed to dry for 1hr. The plates were then scanned by the ELISpot reader (CTL ImmunoSpot^®^ S6 Universal M2 Analyzer, Cellular Technology Limited, Shaker Heights, Ohio) to identify and count spots for analysis.

### METHOD OF CALCULATING THE PERCENT OF MEMORY B-CELLS FOR SPECIFIC ANTIGENS IN EACH ASSAY:

**Figure 2.**
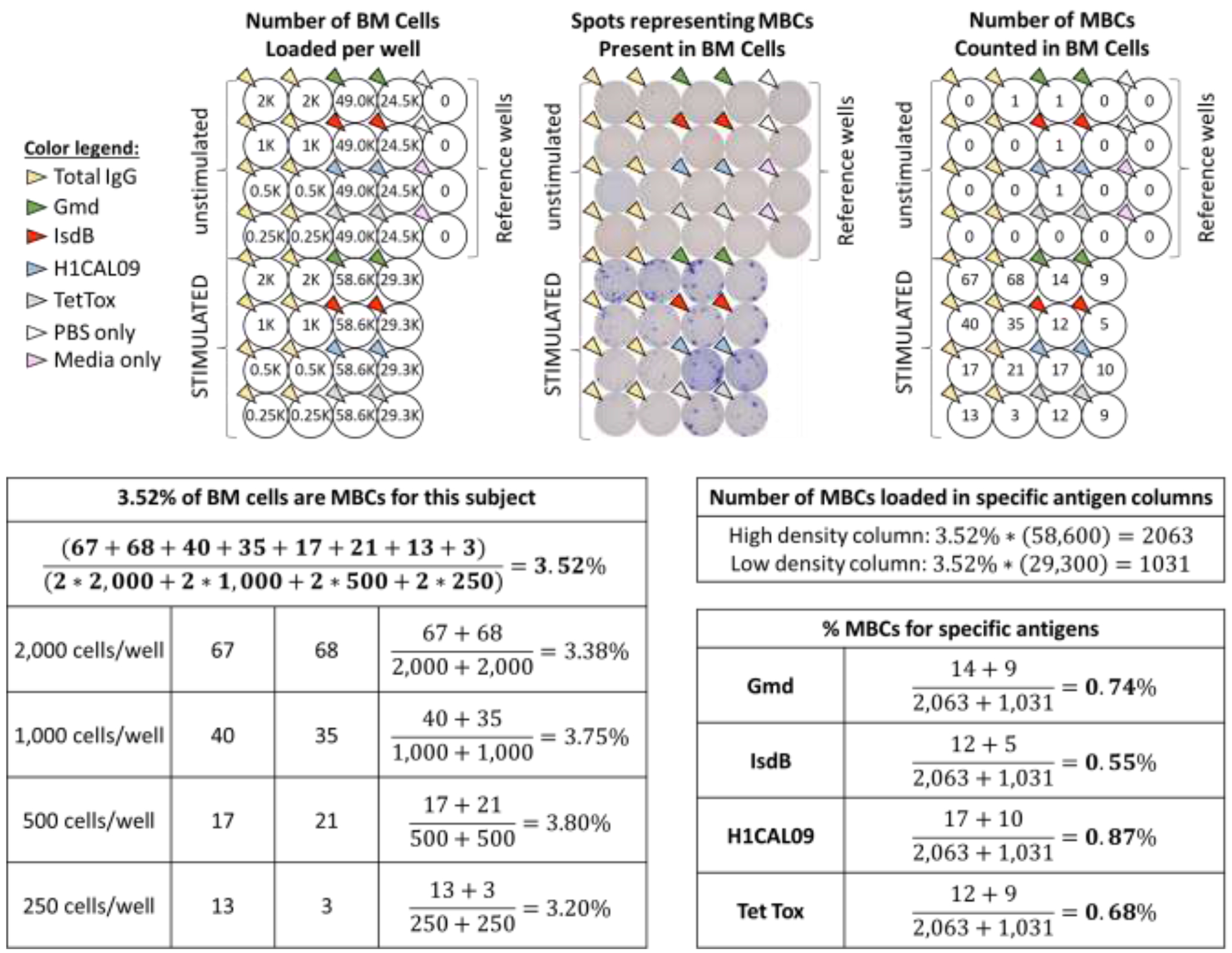
Method of calculating the percentage of memory B-cells (MBCs) responding to specific antigens using an uninfected bone marrow (BM) cell sample as an example. An exampl**e of one uninfected patient’s bone** marrow results is shown here to demonstrate the method of calculation.

The number of spots that were developed in the total IgG wells of each assay represented memory B-cells that were producing total IgG antibodies. Dividing the number of total IgG spots by the number of cells (PBMCs or bone marrow cells) originally loaded into those wells determined the percentage memory B-cells present in the cell sample for each assay (Figure 2). The percentage of memory B-cells was then multiplied by the total number of cells loaded into the wells for each assay to determine the total number of IgG memory B-cells. The number of spots that occurred in the antigen coated wells represents memory B-cells in those wells that are producing IgG antibodies against those specific antigens. The number of spots in these wells was divided by the total number of memory B-cells to determine the percentage of antigen-specific memory B-cells.

### LUMINEX-BASED IMMUNOASSAYS:

IgG antibodies against IsdB and Gmd were measured in serum, blood plasma, bone marrow plasma, and Day 6 antibody-rich supernatant from PBMCs and bone marrow cells via a custom Luminex assay following methodology previously described to determine if a correlation with infection existed ^17,22,23,28,29^. As previously described, after the addition of beads, serum and plasma samples were diluted 1:10,000, Day 6 antibody-rich supernatant samples were diluted 1:2. Concentrations of 27 cytokines were also measured in serum, blood plasma, and bone marrow plasma using three commercially available Luminex assay kits (Millipore Sigma Corporation, Saint Louis, MO) per their established protocols HTH17MAG-14K for 25 Th17 cytokines, HCYTA-60K for IL8, and HCVD3MAG-67K for C-reactive protein (CRP). Samples for Th17 and IL8 were tested without dilution while those for CRP were diluted 1:40,000. All Luminex assays were run on an xMAP INTELLIFLEX^®^ System (Luminex Corp., Austin, TX).

## Results

### ANTI-ISDB ANTIBODY LEVELS CORRELATE WITH INFECTION

**Figure 3.**
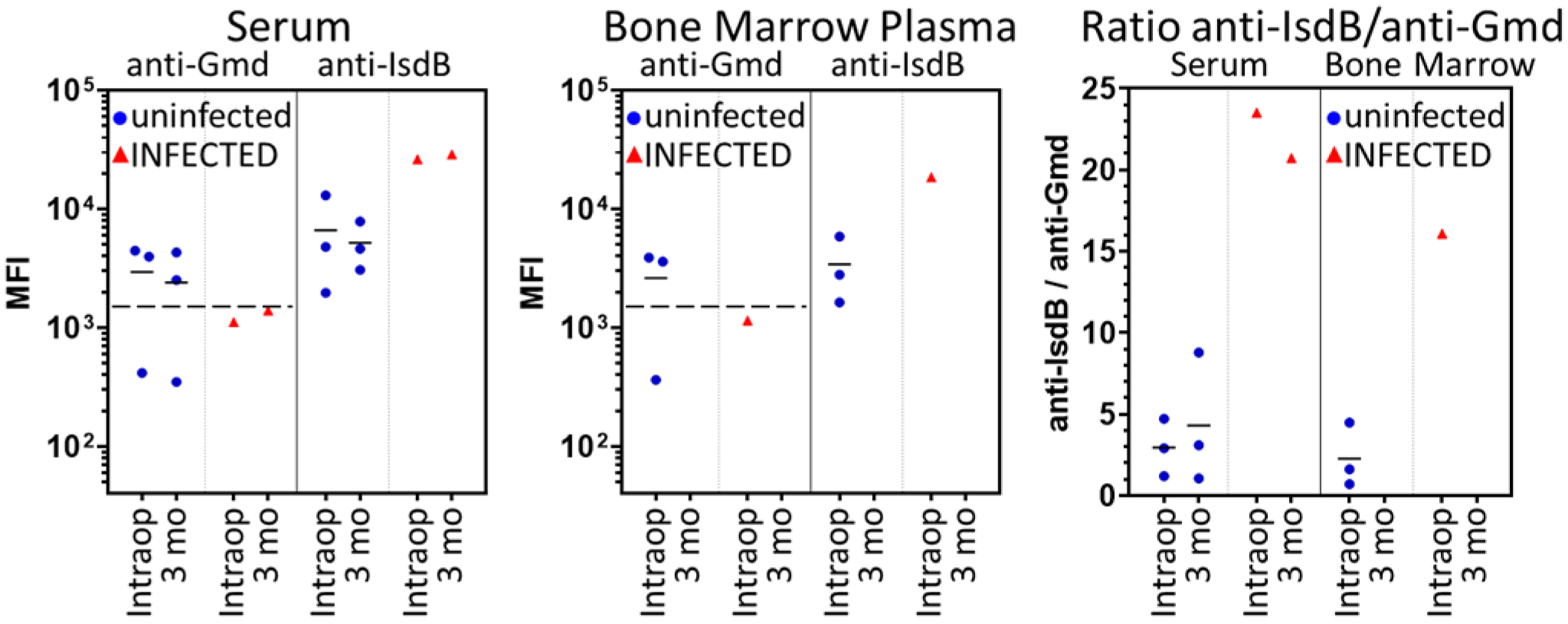
Higher titers of anti-IsdB vs anti-Gmd IgG in serum and bone marrow plasma of both uninfected and infected subjects but more so for the infected. Titers are shown as median fluorescent intensity (MFI) with means (solid lines) and the previously determined physiologically relevant level^17^ (dashed lines).

Serum antibody levels showed a stronger anti-IsdB response than for anti-Gmd in both uninfected and infected subjects, though to a larger degree for the infected subject, which is consistent with our prior studies ^17,21,22^ (Figure 3). This anti-IsdB/anti-Gmd ratio was also seen in bone marrow plasma for the infected subject, but not for the uninfected subjects. The anti-IsdB/anti-Gmd ratio was greater overall for the infected subject. The level of anti-Gmd was low for the infected subject and one uninfected subject. These differences were also detected in serum at three months follow-up. Blood plasma titers were the same as serum (data not shown). This suggests a bias towards anti-IsdB antibodies either predisposes a subject to pathogenic infection or is driven by the infection itself.

Cytokine analysis showed CRP was elevated for the infected subject at the time of surgery in serum, blood plasma (data not shown), and bone marrow plasma, but was reduced to normal levels in serum and blood plasma at three months follow-up (Table 1). CRP in serum and blood plasma for the uninfected subjects was normal at the time of surgery and at three months follow-up. The results from the other 26 cytokines tests did not reveal any remarkable differences (data not shown).

**Figure 4.**
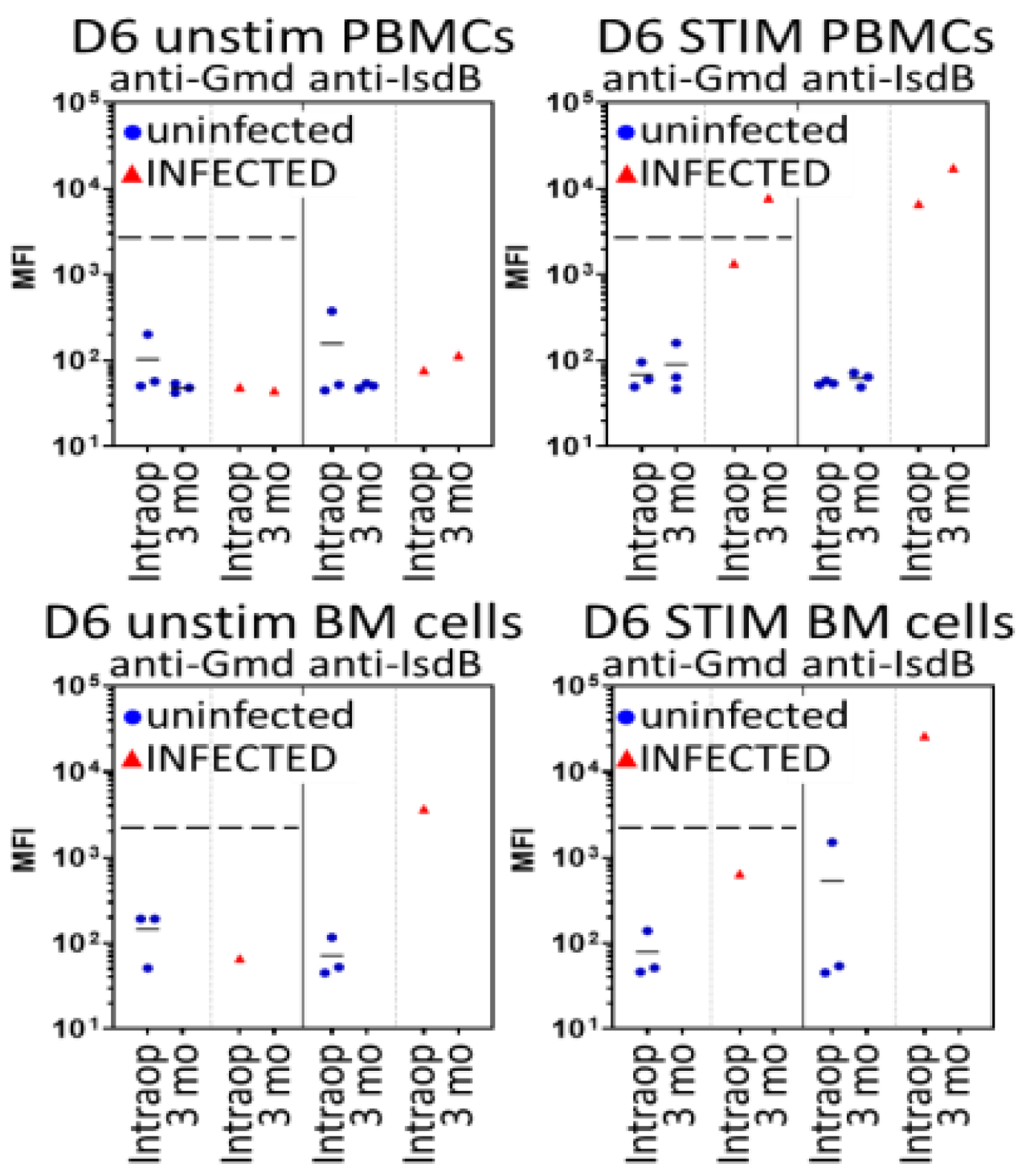
Increased anti-IsdB IgG in Day 6 culture supernatant from bone marrow (BM) cells and stimulated PBMCs from the infected subject. Titers are shown as median fluorescent intensity (MFI) with means (solid lines) and the previously determined physiologically relevant level^17^ (dashed lines).

Luminex tests of supernatant saved from ELISpot cell cultures after six days of incubation from infected bone marrow cells, with and without stimulation, aimed to determine if memory B-cells present systemically in blood and locally in bone marrow would secrete antibodies that would correlate with infection. A strong anti-IsdB vs anti-Gmd response was seen in the infected patient’s bone marrow cells with and without stimulation (Figure 4). This increased anti-IsdB vs anti-Gmd response also existed to a lesser degree in the infected patient’s PBMCs with, but not without, stimulation. In contrast, supernatant from uninfected PBMCs and bone marrow cells, with and without stimulation, had low levels of anti-IsdB and anti-Gmd antibodies, which were not markedly different. This is consistent with the possibility that infection drives IsdB and Gmd specific memory B cells into circulation. The higher titers of anti-IsdB in blood, bone marrow, and supernatant from the infected patient supports the hypothesis of its association with infection.

### ELISPOT DETECTION OF ANTI-ISDB ANTIBODY-SECRETING CELLS CORRELATE WITH INFECTION

**Figure 5.**
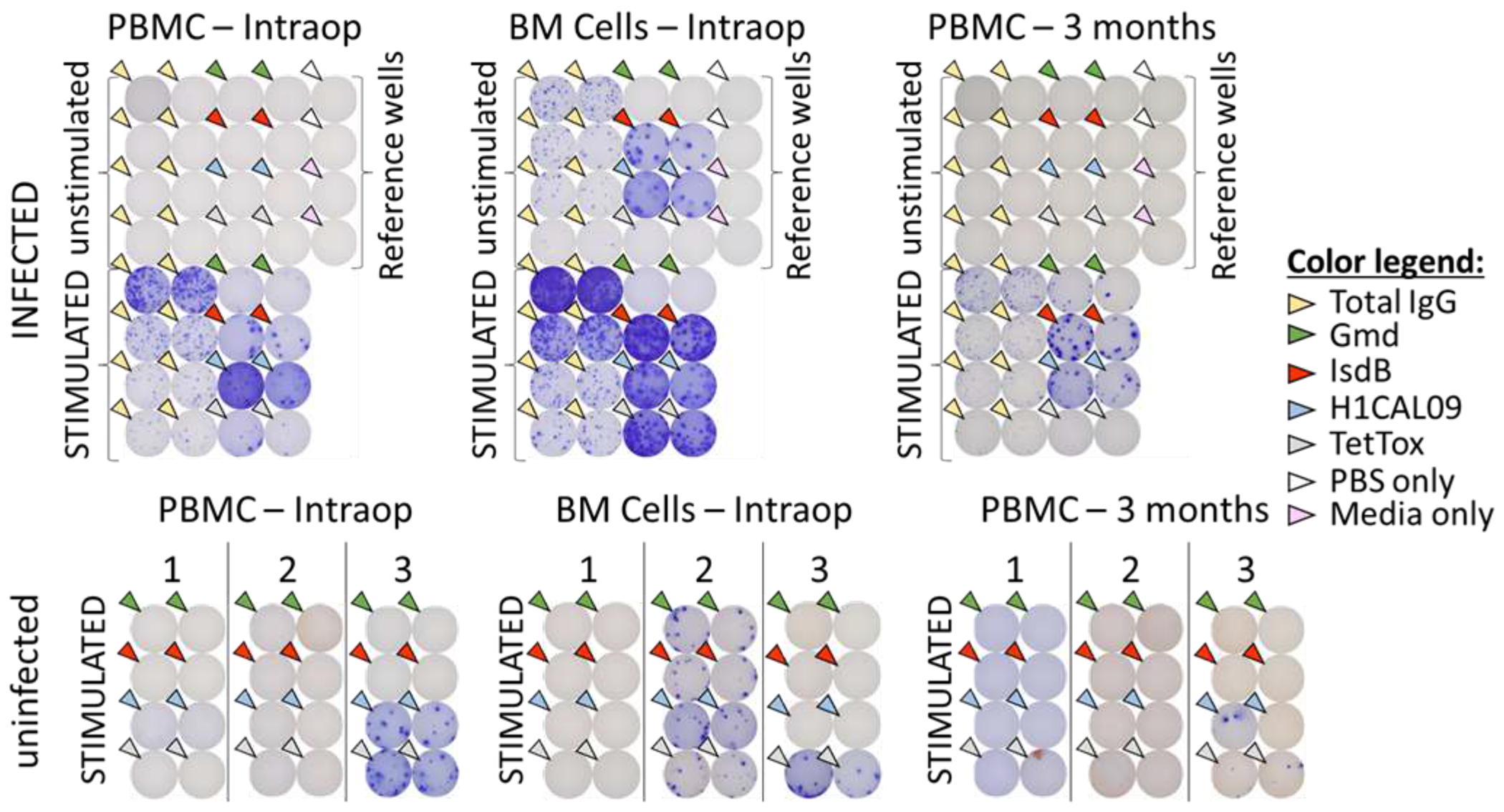
ELISpot assays revealed large numbers of anti-IsdB IgG producing bone marrow cells proximal to a *S. aureus* infected total hip implant. Spots representing anti-IsdB and anti-Gmd antibody-secreting cells systemically in PBMCs and locally in periprosthetic bone marrow cells are shown for the infected patient (top) and the three uninfected patients (bottom).

To determine whether antibody responses are due to circulating and/or localized memory B-cells, ELISpot assays were performed to enumerate antibody-secreting cells specific to IsdB and Gmd. Assays without stimulation were used to reveal existing antibody-secreting cells. Stimulation assays were used to differentiate existing memory B-cells into antibody-secreting cells. A qualitative comparison of spot accumulation in ELISpot images for the infected subject showed the presence of anti-IsdB antibody-secreting cells in bone marrow with and without stimulation but no detection of anti-Gmd antibody-secreting cells (Figure 5). For the infected subject’s PBMCs, no spots were detected without stimulation. With stimulation of infected PBMCs, anti-IsdB antibody-secreting cells were detected at the time of surgery and three months later, while anti-Gmd antibody-secreting cells were barely detected at the time of surgery but were more evident three months later. For the uninfected subjects, no antigen specific IgGs were detected for unstimulated PBMCs or bone marrow cells (images not shown). PBMCs and bone marrow cells from two uninfected subjects with stimulation showed no anti-IsdB nor anti-Gmd responses. For one uninfected subject, bone marrow cells with stimulation showed similar anti-IsdB and anti-Gmd responses.

A quantitative assessment of spot counts in the ELISpot images enumerates what was observed qualitatively, further supporting the correlation of anti-IsdB with infection. Without stimulation, 0.43% of bone marrow memory B-cells for the infected patient were existing anti-IsdB antibody-secreting cells; this increased to 1.13% with stimulation (Table 2). Anti-Gmd percentages for bone marrow could not be calculated for the infected patient due to lack of spots. For the infected patient’s PBMCs, no spots were detected without stimulation and percentages could not be calculated. With stimulation of infected PBMCs, anti-IsdB antibody-secreting cells were calculated as 0.07% of memory B-cells at the time of surgery and 0.48% three months later. Anti-Gmd antibody-secreting cells in stimulated PBMCs from the infected patient were 0.01% of memory B-cells at the time of surgery and increased to 0.11% three months later. For the uninfected subjects, spots were only detected for bone marrow cells with stimulation from one uninfected subject and showed similar percentages with 0.55% of memory B-cells as anti-IsdB antibody-secreting cells and 0.74% of memory B-cells as anti-Gmd antibody-secreting cells. Summarizing, while the infected subject had evidence of circulating and bone marrow resident memory B-cells against both IsdB and Gmd, the uninfected subjects had no IsdB or Gmd-specific memory B-cells in circulation and only one had memory B-cells in the bone marrow at the time of surgery which were balanced against the two antigens.

## Discussion

To develop effective vaccines and passive immunizations against *S. aureus*, investigators have been cataloging all the known antibodies against this pathogen to define the immune proteome^19,20^. By studying the functional interactions of these antibodies with bacteria and immune cells in vitro and in animal models^30^, combined with epidemiological data^17,31,32^, including serum levels in patients over the course of *S. aureus* infection and clinical outcomes, their protective and pathogenic value is being established^33^. Our osteomyelitis research is an example of this, in which we identified anti-Gmd antibodies as protective^15–18^ and anti-IsdB antibodies as pathogenic^12,17,21^. Consistent with this hypothesis, we detected a high frequency of IsdB-specific antibody-secreting cells (plasmablasts) in the periprosthetic bone marrow of an infected subject, even without stimulation. Furthermore, after polyclonal in vitro stimulation, the number of IsdB-specific antibody-secreting cells increased, demonstrating the infiltration of memory B cells specific for IsdB in the infected bone marrow. These observations suggest antigen-driven recruitment and retention of B cells to^34^ make IsdB-specific antibodies. Indeed, our observations are reminiscent of studies using influenza viruses, which show that the expansion of antigen-specific plasmablasts and plasma cells is often associated with adverse disease outcomes rather than protective immunity^35,36^.

It is also remarkable that anti-Gmd antibody-secreting cells were undetectable at the time of surgery in periprosthetic bone marrow for this infected patient. This suggests that the ratio of IsdB to Gmd antibodies may be important, and Luminex results showed that this infected patient had very high titers of anti-IsdB antibodies and low titers of anti-Gmd antibodies at the time of surgery in sera, plasma from blood and bone marrow, and in Day 6 supernatant from bone marrow cells with and without stimulation, and PBMCs with stimulation. Three-month follow-up data showed that while CRP in the infected patient had returned to normal, no change occurred in the high anti-IsdB/anti-Gmd ratio, suggesting that this patient could not correct the unfavorable immune proteome. Notably, this patient required implant removal, antibiotic therapy, and reimplantation before full recovery.

For the uninfected patients, anti-IsdB and anti-Gmd-producing cells were only detected in stimulated bone marrow cells for one patient and were present at comparable frequencies. Consistent with our results on healthy controls in other studies^17,21^, Luminex results from the uninfected patients showed anti-IsdB greater than anti-Gmd in serum and blood plasma, though the difference was to a lesser degree than for the infected patient. Furthermore, no difference was seen between anti-IsdB and anti-Gmd titers in bone marrow plasma or Day 6 supernatant from the uninfected patients, consistent with the absence of infection.

A major limitation of this study is that we had only one infected and three controls who were healthy at the time of sampling. Caution is therefore necessary in extrapolating the significance of these results and cannot be extrapolated to all infected subjects without further studies. Thus, larger prospective studies to test this with statistically powered cohorts remain an important future direction. Nonetheless, our data are consistent with several other observations, indicating that antibody responses to IsdB are associated with infections and complications after joint replacement surgery, which supports the Immune Proteome hypothesis. In addition, it will be important to determine whether differences in immunity to IsdB and Gmd are pre-existing prior to surgery or reflect a process that occurs during or after the surgery. Collectively, these case reports demonstrate the utility of ELISpot assays, combined with more conventional antibody measurements, to assess local vs. systemic antigen-specific humoral responses in total hip arthroplasty patients and support a larger study to test the hypothesis that anti-IsdB producing cells proximal to *S. aureus* infected implants are associated with recalcitrant infections and septic death.

## Conclusions

A human immune proteome against *S. aureus* exists in which antibodies against IsdB appear to be pathogenic, while antibodies against Gmd appear to be protective. While circulating antibodies against *S. aureus* exist in periprosthetic joint infection patients, the protective vs. pathogenic effects of antibodies produced in the local environment may be more impactful to the patient’s clinical outcome.

## Figures and Tables

**Table 1:** Elevated CRP in serum and bone marrow plasma from the infected subject at the time of surgery that returned to a normal level at three months post-op. CRP levels were determined by Luminex (note < 2 mg/dL is considered normal).

		**CRP (mg/dL)**	
		**Intraop**	**3mo.**
**Serum**	**INFECTED**	14.9	1.7
	*Uninfected*	*0.8, 1.7, 0.8*	*0.8, 2.1, 0.9*
**Bone Marrow Plasma**	**INFECTED**	15.9	--
	*uninfected*	*0.8, 1.5, 0.8*	--

**Table 2.** Percentages of anti-IsdB vs anti-Gmd memory B-cells (MBCs) in PBMCs and bone marrow (BM) cells with and without stimulation. The ELISpot images in Figure 5 were quantified, and the percentages were calculated as described in Figure 2. Dashes indicate instances when no spots were detected; thus, percentages could not be calculated.